# Science for tomorrow’s neurosurgery: insights on establishing a neurosurgery patient group focused on developing novel intra-operative imaging techniques

**DOI:** 10.1186/s40900-024-00649-0

**Published:** 2024-10-29

**Authors:** Oscar MacCormac, Matthew Elliot, Lisa Whittaker, Anisha Bahl, Silvère Ségaud, Andrew J. Plowright, Shannon Winslade, Alice Taylor-Gee, Bella Spencer, Tom Vercauteren, Jonathan Shapey

**Affiliations:** 1 School of Biomedical Engineering and Imaging Sciences, King’s College London, Lambeth Palace Road, London, SE1 7EH UK; 2 Dept. of Neurosurgery, King’s College Hospital, Denmark Hill, London, SE5 9RS UK; 3 The Brain Tumour Charity, Hanger Green, London, W5 3EL UK; 4 The University of Manchester, 1.006 Carys Bannister Building, Manchester, M60 1PL UK

**Keywords:** Patient involvement, Public engagement, PPI, Neurosurgery

## Abstract

**Background:**

Incorporating patient and public involvement (PPI) in research is crucial for ensuring the relevance and success of studies, yet it remains significantly underutilised in surgical research.

**Main body:**

This commentary presents insights from our neurosurgical research team’s experience with establishing and working with a PPI group called “Science for Tomorrow’s Neurosurgery” on research regarding novel intra-operative optical imaging techniques. Through collaboration with patient-focused charities, we have successfully incorporated patient perspectives into our work at each stage of the research pipeline, whilst adhering to core PPI principles, such as reciprocal relationships, co-learning, partnerships, and transparency.

**Conclusion:**

We highlight the specific value added to our work in terms of participant recruitment, research ethics and results dissemination.

## Abstract (plain language summary)

Including patients and members of the public in medical research is really important. It helps make sure the research is useful and successful. Surprisingly, it’s not used enough in surgical research. This article talks about how our team of brain surgeons and scientists worked with a group called “Science for Tomorrow’s Neurosurgery” to include brain surgery patients in our research about new ways to look at the brain during surgery. We worked closely with charities that focus on helping patients with brain conditions, and we made sure to listen to what patients had to say every step of the way, from planning our research to sharing our results. We followed some important rules, like making sure everyone involved felt like they were being treated fairly, learning together, working together with different groups, and being honest and open. We found that having patients involved in the design and development really helped us find the right people to take part in our research, make sure we were doing things the right way, and share our findings in a way that made sense to everyone.

## Background

Funding for neurosurgical research has continued on an overall upward trend over the past 20 years [1, 2], correlating with an increase in high quality studies [3] and an ever increasing focus on digital healthcare technologies [4]. The importance of patient and public involvement (PPI) in research is becoming increasingly more evident [5–9]. This is reflected by its inclusion in the pre-clinical (IDEAL 0) phase of the IDEAL framework [10] for new healthcare technologies and device development.

However, incorporation of PPI into surgical research has been reported to be as low as 1.7% [11, 12], compared with up to 44.5% across other specialties [13]. This is surprising given that PPI has been formally included in the UK National Institute for Health Research (NIHR) research guidelines [14] and is now a requirement for many research funding bodies [15, 16].

Our research team founded a PPI group called “Science for Tomorrow’s Neurosurgery”, focused on supporting studies developing novel intra-operative imaging techniques in neurosurgery. The group included patients with first-hand experience of neuro-oncology and neuro-vascular surgery, providing a range of perspectives pertinent to our work. The technology in question is ’hyperspectral imaging;’ a technique that involves a special type of camera, already used in other industries that has the potential to see more than our most advanced cameras can see. We hope to harness the additional information captured to help identify healthy and unhealthy tissue in a much more timely and clear way, resulting in safer, more precise surgery in the future [17]. The first in-human use of a lightfield hyperspectral imaging system [18] is a strong example of a study supported by this group. The group included patients with first-hand experience of neuro-oncology and neuro-vascular surgery, providing a range of perspectives pertinent to our work.

By reporting our positive experience of fully integrating PPI into our research cycle, we aim to provide a blueprint for colleagues in other surgical specialties to incorporate PPI more readily in their work.

We have structured this commentary on five areas we have identified where there were salient learning points for us during our PPI experience: Participant Recruitment, Funding, Maintaining Engagement, Value Added and Wider Engagement.

## Participant recruitment

When forming a PPI group, it is important to recruit a diverse range of participants reflecting relevant past healthcare experiences and the socioeconomic, educational and cultural backgrounds that the research aims to impact.

Our research focuses on novel intra-operative imaging methods for neurosurgery, particularly in patients with brain tumours, arteriovenous malformations (AVMs) and brain aneurysms. We focused our recruitment of PPI group members on people with these conditions. We also extended the invitation to join the group to patient carers and relatives as they can often provide very useful insights from a different perspective.

**Fig. 1:**
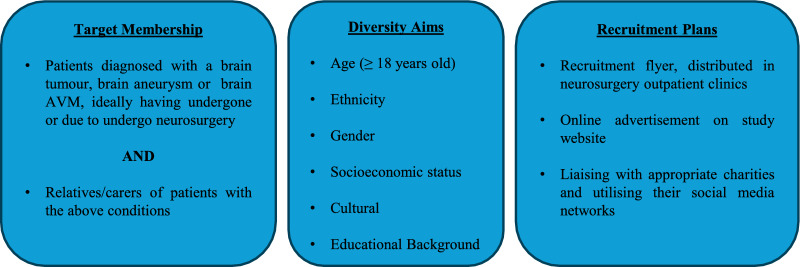
PPI recruitment summary for “Science for Tomorrow’s Neurosurgery”

To achieve these recruitment goals, first we appointed a PPI chair; a brain tumour patient with experience in both research and involvement activities, with whom we connected via The Brain Tumour Charity. We then advertised in the neurosurgery outpatient clinics at King’s College Hospital, U.K. King’s is our local neurosurgical centre and thus all potential patients recruited to our research trials will come through this centre. We chose to advertise here because we hoped we were reaching a population representative of that served by the hospital. Unfortunately, due to the timing of the group’s foundation coinciding with the COVID-19 pandemic, there were very few face-to-face appointments happening in hospitals at that time. We thus sought other methods to recruit participants. One of the most fruitful was to seek support from relevant charities, such as The Brain Tumour Charity, The Butterfly AVM Charity, Headway and The Brain Charity, who were able to grant us access to their social media and patient involvement networks, from which the majority of the “Science for Tomorrow’s Neurosurgery” group were recruited. Whilst we recognise that these methods may introduce bias [19], given the challenging circumstances presented by the COVID-19 pandemic, it was necessary to recruit via these methods. Our recruitment process is summarised in Fig. 1.

Interestingly, the majority of group participants were recruited from outside of the research site catchment area, ensuring that a range of experiences and insights could be offered from patients that had engaged with a number of different neurosurgical teams, whilst reducing the risk of recruitment bias from those that may have had a positive experience with our clinical team in the past. Indeed, the only group member who had undergone surgery undue our site’s neurosurgical team in the past had a difficult post-operative experience with long lasting side effects. When exploring this participant’s motivation to join the group, it was to partake in and advise on research that could result in safer surgery in the future, so that their experience was not repeated.

The difficulty with being limited to an electronic recruitment method was that we found it restricted our ability to ensure diversity, particularly amongst underrepresented groups, within our PPI group. This is particularly relevant as the immediate local population to the research site is 35% Black African/Black Caribbean or Black British with an average age of 33.7 years [20, 21], whereas the wider area served by the site is 91.3% white (89.1% White British) with an average age of 41.7 years.

We had discussed the possibility of electronically screening for ethnic/cultural/socioeconomic background as part of the recruitment process with our PPI chair, however we decided that this risked introducing a bias to our selection. As well as this, we had budgeted for a maximum group size of 7 patient participants, meaning we risked turning respondents away based on their background, which may discourage them from taking part in future PPI activities. We did not feel this would be appropriate.

We have expanded further on diversity within our group in 4.3, Table 1.

## Funding

Funding was sought from a number of different sources, primarily the NIHR Invention for Innovation (i4i) grant scheme, Wellcome Trust Innovator Award and core Wellcome/EPSRC funding for the King’s College London Centre for Medical Engineering. The initial budget, included in the NIHR i4i grant, was £12,100 to cover PPI costs over a 3 years project. Costs were calculated based on two in-person meetings per year, which included both engagement and involvement activities. Specifically, this comprised £900 patient consultancy fee per meeting (£5400), £500 per meeting for an independent facilitator (£3000), £500 per session for a live illustrator to capture the themes of the meetings (£3000), £50 per year for review of key documentation (£150) and £550 for a patient representative to attend a conference to present the work. In hindsight, whilst this was adequate to cover the costs for our *involvement* work, it was not adequate to cover *engagement* projects, such as the Illuminating Brain project, discussed in Sect. "Wider Engagement". Incorporating an engagement activity into our work programme became a particular priority due to the difficulties in arranging in-person involvement meetings, largely as a direct result and impact of the COVID-19 pandemic, but also due to patient factors limiting their ability to travel This activity required a budget of £6,600. In future, we would plan for both involvement and engagement activities within a single budget application.

## Maintaining engagement

Maintaining engagement with a PPI group is essential to ensure ongoing, meaningful contributions to research throughout the duration of a study, which often require input over years rather than short periods of time. To achieve this, we used the principles outlined in the Patient-Centred Outcomes Research Institute (PCORI) Engagement Rubric for Applicants: *Reciprocal Relationships*, *Co-learning*, *Partnerships* and *Transparency, Honesty and Trust* [22].

### Reciprocal relationships

In order to achieve a reciprocal relationship between all stakeholders we allocated a patient chairperson for the group. The chairperson was recruited with the support of the Brain Tumour Charity using its existing involvement network. This enabled us to work closely with a patient participant already experienced in PPI in research. This is a very valuable first step, especially for teams that are new to PPI, and has been demonstrated to be an effective way of maintaining engagement [23]. We drew on the chairperson’s experience to formulate the frequency of group meetings, review meeting agendas and to ensure that research questions/decisions put forward to the group were appropriate. At the same time we ensured there was dedicated time for meaningful feedback and discussion, as well as time for the patient group to speak about their own lived experiences.

Appointing an independent group facilitator was crucial to the success of each meeting by assisting in developing the meeting agendas and ensuring that the aims of each meeting were met. Having an independent facilitator moderate discussion also empowered patient participants to speak and contribute throughout the process. The facilitator also served as a single point of contact for group members, which is important for maintaining relationships, facilitating a direct line of communication between the core research team and the PPI group participants outside of the group meeting environments, consistency and ensuring group values are adhered to throughout [23, 24].

Our success in maintaining a reciprocal relationship is exemplified by the following quote from our patient chairperson

The first patient involvement group meeting was a great success, with a group of very interesting people from various walks of life watching some informative presentations and asking some fantastic questions. All of the comments and questions arising from this session will help us to better understand the needs and concerns of patients and enable us to make sure that we address these issues in our communications for this project.

and quote from our senior clinical researcher

The PPI session was an invaluable way to explore important clinical and research questions with a range of neurosurgery patients. The feedback received will help us shape our research ensuring that it remains patient-centred.

### Co-learning

It is important to ensure that the research team does not try and turn patient participants into researchers [22], but rather empower them to direct the research questions being answered. This is because the value of PPI is in providing a unique, patient-focused perspective to research questions that may not otherwise have been considered [25]. For this process to be effective, PPI group participants need to understand the research process and the study itself whilst ensuring that the values important to them are upheld and considered throughout. Therefore, during our group meetings, research team members gave three 15 min presentations on specific technical aspects of the study as well as proposed research methodology, targeted to a lay audience. Following these presentations, ample time for discussion, feedback and questions was provided, guided by our group facilitator. We have found this to be very successful, exemplified by the following quotes our group members:

The quite complex information was presented in such a way that it was easily understood by those of us with a non-medical background. The information was pitched at exactly the right level and was interesting, informative and of great interest. It also made me feel as though I was actively involved in helping people in the future. I have some fairly difficult side effects to live with since my op[eration], so knowing that it may be possible to limit or eradicate those in the future makes me feel as though I have done something to help others. Living with any type of brain tumour can be quite life changing and by being involved with the research it will hopefully benefit others in the future. It was also great to meet other people who have been in the same situation, to meet the researchers that are working towards better outcomes for patients and to have some insight as to how tumours are seen by surgeons. I found it particularly fascinating to see how AI can make the colour spectrum appear the same to each and every surgeon rather than relying on an individual’s interpretation of the colours they see themselves, which can differ greatly from person to person.

It was a very professional and friendly group with people from many different walks of life who were all made to feel that their own views were equally important to the research. I would like to take this opportunity to thank you all for allowing me to be a part of this very important research project and a huge thank you for being able to present what can be very complex information in a way that could be understood by all.

In turn, patient participants provided valuable input at each stage of the research, with particular insights given to the patient facing documents and study recruitment pathway. One such example was determining when best to approach potential study patients. Understanding the lived experience from our patient group and the hugely overwhelming experience of being told they had a brain tumour that required surgery helped shape our recruitment pathway in that we did not approach patients during this clinic appointment, but simply asked their treating clinician to request if a member of the research team could contact them at a later date to discuss potential studies. This was very valuable in ensuring our research did not contribute negatively to an already difficult encounter.

**Fig. 2:**
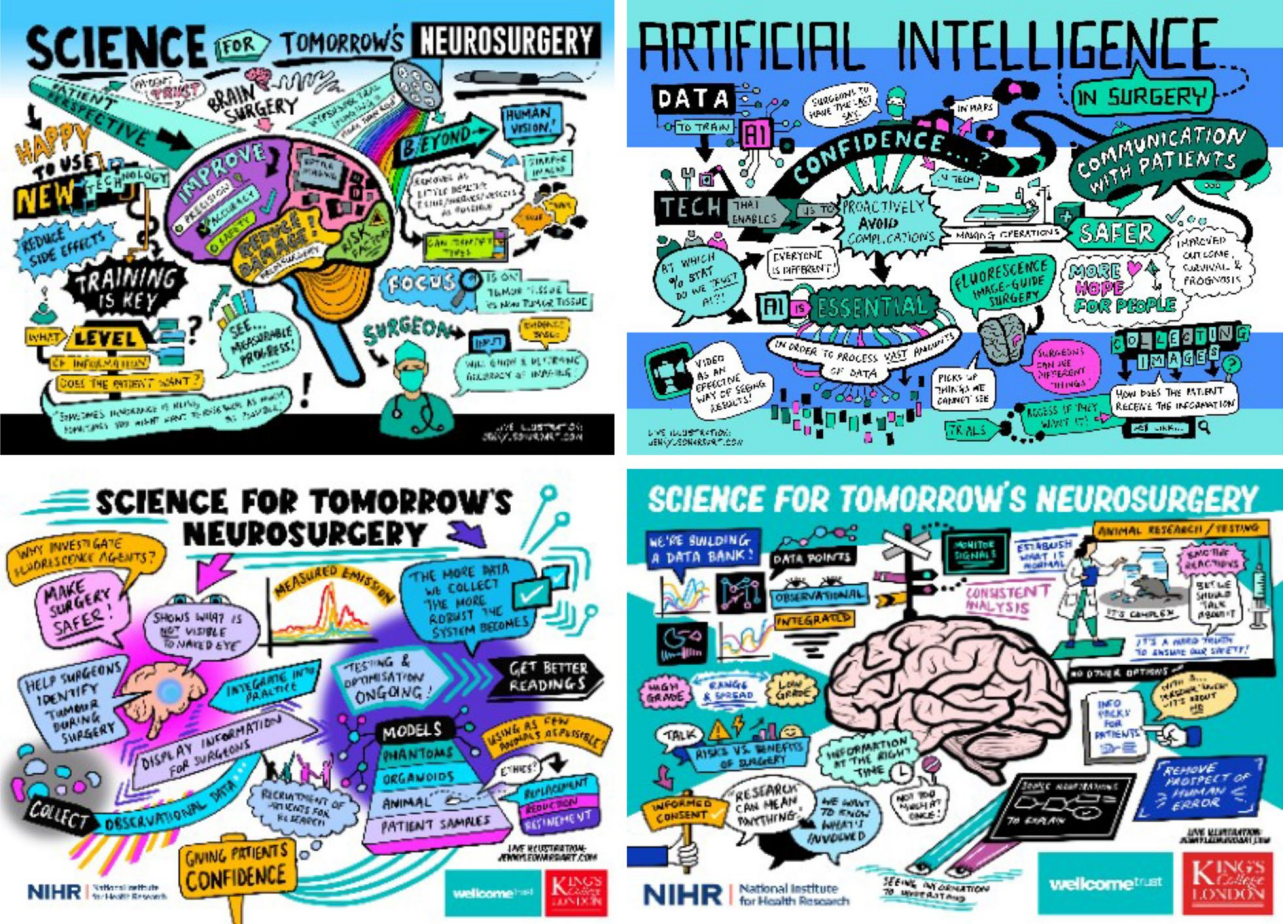
Results from live illustrations during four meetings of our Science for Tomorrow’s Neurosurgery PPI Group meetings. Illustrations courtesy of Jenny Leonard, https://jennyleonardart.com

To consolidate the learning points from each session, we collaborated with a freelance artist who creatively captured the discussions using live illustrations. Illustrations have long been shown to be an effective method for consolidating learning [26] and each illustration has been received positively by all group members. Examples of the illustrations are shown in Fig. 2

### Partnerships

This principle emphasises the importance of valuing the time and contributions from the patient group, ensuring diversity within the group and making appropriate provisions for group members where necessary [22]. Time should be financially compensated, as should any expenses incurred to be partake in meetings/events. NIHR UK suggest a consultancy fee of £25/hr for each patient participant [27]. We have summarised our PPI group demographics in Table 1.

It was also important to consider accessibility, particularly with a neurosurgical patient group spread across the UK, some of whom experience physical disabilities. This prompted the decision, made jointly with the PPI chair, to continue with primarily online meetings, even with the height of the COVID-19 pandemic subsiding, allowing this vulnerable group of participants to still feel connected to each other and learn about the latest technology whilst staying safe in their own homes. Other additional events that we have run have been planned well in advance, ensuring venues are appropriately accessible to all participants.

**Table 1:** Summary of Science for Tomorrow’s Neurosurgery participants by age, sex and ethnicity

Age (years)	N
18–25	1
26–45	3
46–65	2
66+	1
**Sex**	
F	5
M	2
**Ethnicity**	
White (not specified)	2
White British	4
White African	1

### Transparency, honesty and trust

These final principles require all parties to ensure open and honest communication throughout the course of the research process, including in key decisions that may affect the progress or dissemination of the work [22]. By ensuring regular meetings, as well as having the single point of contact via our facilitator, we ensured all parties were regularly updated with an open channel of communication between the clinical research team and the patient participants. As well as this, at the beginning of the meetings, there was always allocated time for introductions, conversation and the chance for members to reconnect. This worked well within our group and, over time, everyone felt confident to discuss and receive feedback, even when discussing the most challenging and emotive topics such as the need for animal studies to support our research.

## Perceived impact

Many publications have indicated that quantifying the specific impacts of PPI on research studies is challenging [5, 7, 28] and some have even suggested that we should stop trying to measure it [29]. It has been suggested that public involvement should be conceptualised as a two way discussion between participants and researchers, with learning on both sides [30]. This learning is subjective and therefore difficult to quantify, with the risk that attempting to rigorously measure the impact can result in negating the ethical reasons for having PPI in the first place [29, 31, 32]. However, given the reported low degree of patient involvement in surgical research studies [11], we have decided to highlight three areas where there has been clear, specific impact on our work; Ethics, Study Recruitment and Dissemination.

### Ethics

During the course of our work, we have demonstrated that the novel intra-operative imaging system we are evaluating is capable of providing potentially clinically significant results with regards to real-time brain oxygenation visualisation. However, it has become apparent that in order to validate these results and ensure that they are meaningful, we would need to perform a live animal study under general anaesthetic. Conducting scientific research on animals is, understandably, a heavily debated topic [33], with the majority of Britons surveyed now in opposition to animal testing, albeit with some variability depending on the precise nature of the research [34]. This was not the case 15 years ago, with previous results suggesting overall support in the context of medical research [33]. Animal research is therefore not a decision to be taken lightly.

The PPI group proved invaluable when preparing our submission to the Animal Welfare and Ethical Review Body (AWERB), providing commentary on our proposed protocol. Our PPI group discussion also supported our position that a live animal study was necessary, there were no other clear options available to us to validate our work, and that our research was significant enough to justify the study. This input and overall support ultimately facilitated AWERB approval, as well as providing reassurance to the clinical research team that this was an appropriate step to take.

### Study recruitment

When designing our recruitment pipeline for brain tumour patients, we had initially planned to recruit patients directly from the neuro-oncology clinic. We presented this pipeline to our PPI group, who advised us that an improved pipeline would be for the clinician reviewing the patient to ask the patient if they would be happy to be called at a later date by a member of the research team, followed by contacting the patient by phone more than 24 h later if they agreed. This came about from each member of our group describing the first clinic appointment before surgery with phrases such as an “information overload”, and “not able to take it all in”. The PPI group gave input into all patient facing documents, for example recruitment pamphlets, participant information sheet (PIS) and the consent form, ensuring that each of these materials remained relevant, easy to understand and patient focused. This adapted pipeline, along with ensuring patient input in to each stage of the recruitment pathway, may well have contributed to our high (97%) recruitment rate from those approached.

### Dissemination

When applying for study approval from the Health Research Authority (HRA), the Research Ethic Committee (REC) questioned the way we proposed to disseminate our results. Our original plan was to disseminate our results collectively on the study website (https://cai4cai.ml/neurohsi/), however the REC felt it more appropriate to individually post the results to each study participant. We discussed this with our PPI group who felt that they would actually prefer the receipt of results to be optional and thus we could use this as a rebuttal to the REC comments. The REC accepted this rebuttal and allowed us to continue with our planned approach. This emphasises the importance of involving patients at each stage of research design; try as we might to do the best by patients, what we think may be the best approach may not always be seen as such by those with the relevant lived experiences.

## Wider engagement

Building on the success of our PPI group“Science for Tomorrow’s Neurosurgery”, we sought to share our work more broadly and gain additional patient/carer insight (engagement). Art and creative activities have been demonstrated to be effective ways of engaging the public in research, as well as broadening the diversity of contributors [35] and therefore we decided to explore this with a project called “Illuminating the Brain”. This project involved a collaboration between a locally acclaimed graphic designer, members of our research team and involvement champions from The Brain Tumour Charity. All involvement champions are young adults with lived experience of a brain tumour diagnosis and subsequent brain surgery, or young adults with experience caring for a friend or relative with a brain tumour diagnosis. The designer visited a live demonstration of our technology in our state of the art mock operating theatre, followed by an online workshop at which all parties were introduced and themes were identified, before everyone came together in a creative in-person workshop. The workshop involved small group exercises where combinations of patient participants and the clinical research team discussed topics such as “what do I want people to know” and “I wish people could understand...”. Individual exercises included writing down, anonymously, some examples of “the challenges I face”. These topics were chosen by the graphic designer following our introductory meeting and demonstration. Further exercises involved sharing a 5 min story with the group, with the option of bringing an object to support this story. Finally, each participant was invited to “colour me a story”, a free drawing exercise in which participants were asked to draw how the themes of colour and light (key aspects of our research work) were relevant to their journey. This was recorded and all of this information was used to create a piece of art, which was displayed for the first time at Science Gallery London, as part of the “Illuminating the Brain Showcase”.

### Illuminating the brain showcase

**Fig. 3:**
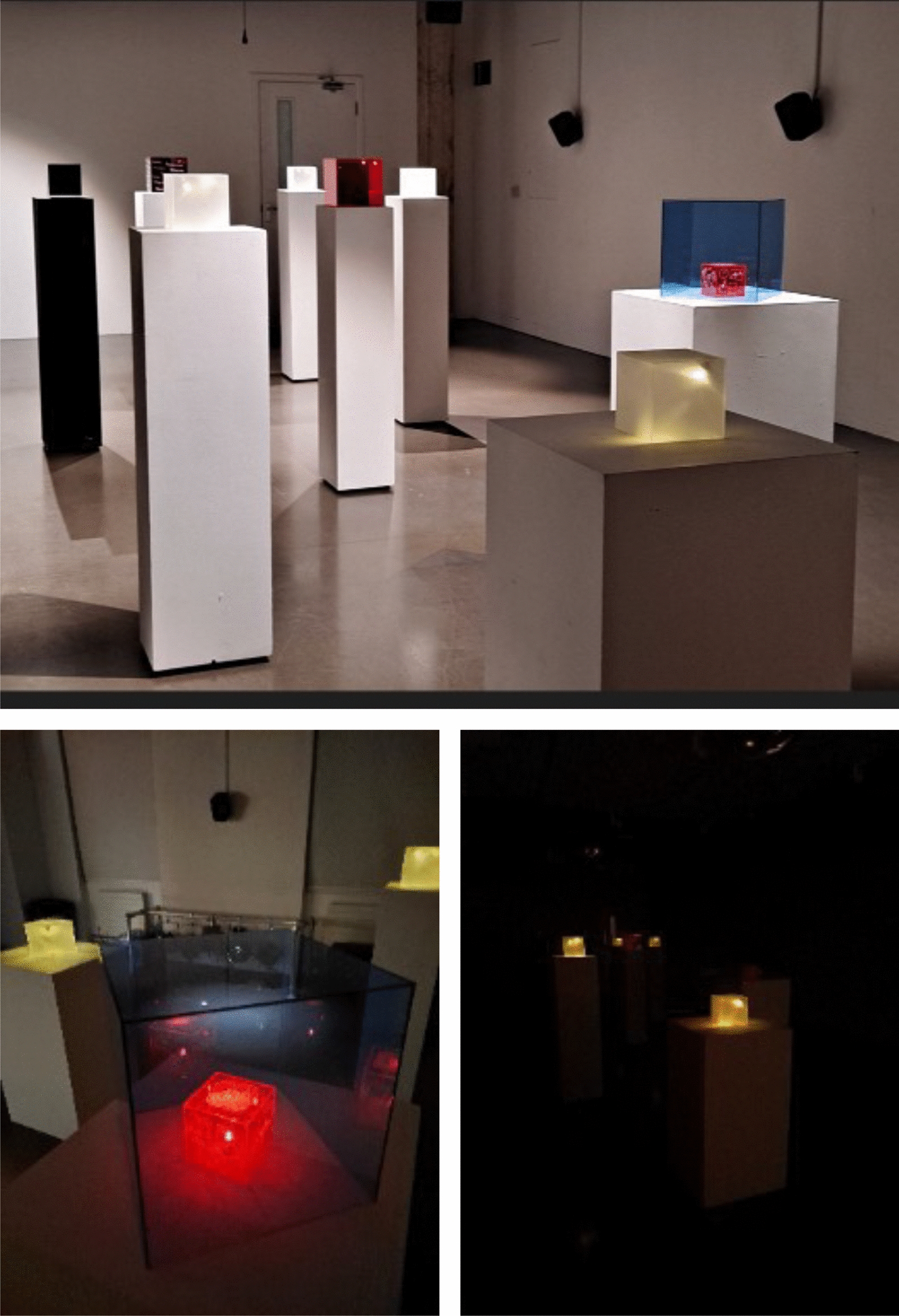
Top: Illuminating Brain Project artwork displayed at The Science Gallery. Bottom Left: Close-up view of individual pieces. Bottom Right: Illuminating Brain Project art displayed in darkness, to emphasise the contribution of colour and light to the project

**Fig. 4:**
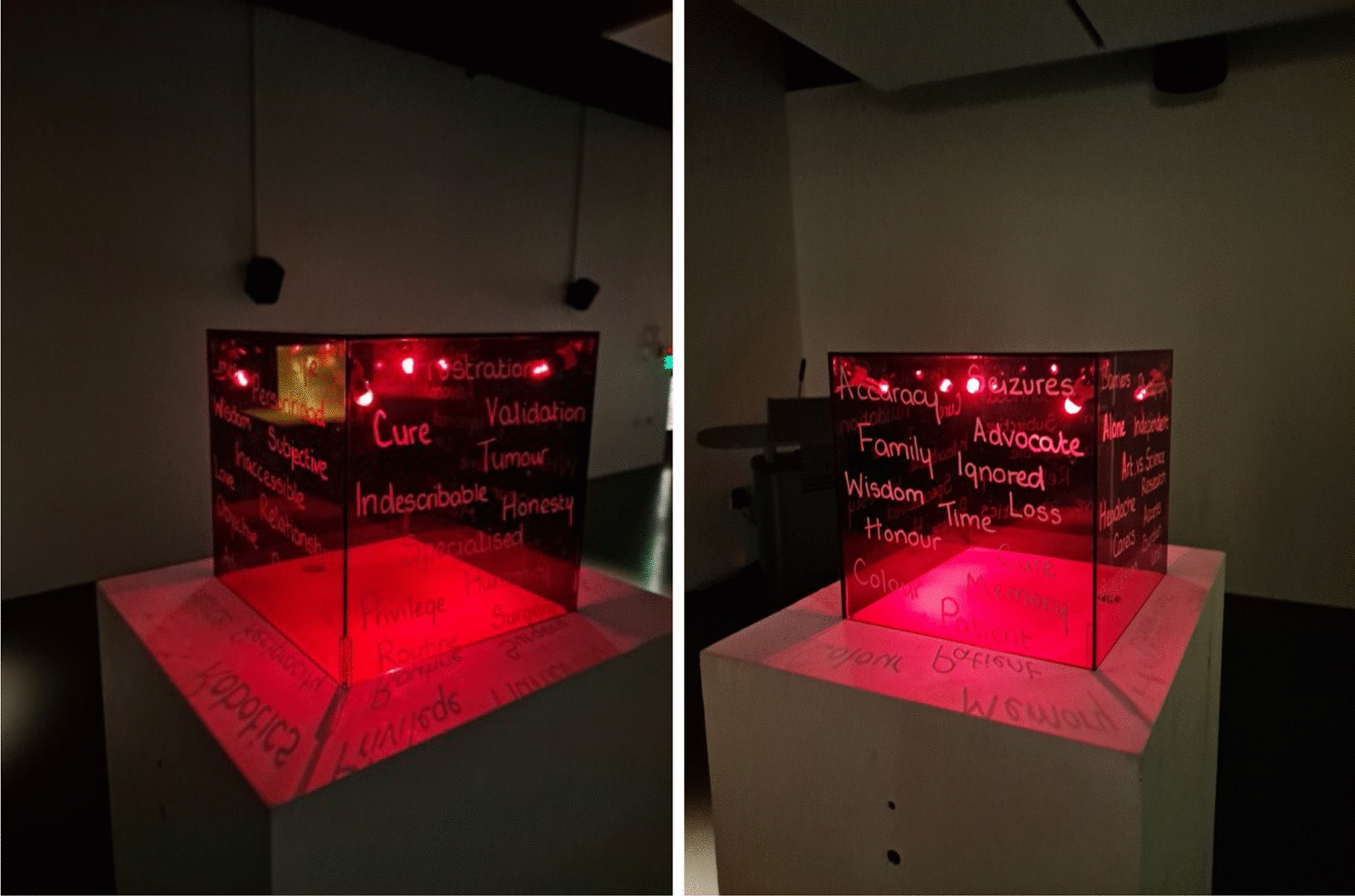
Close up views of an individual piece from the Illuminating Brain Project, demonstrating words and themes from patients and researchers during the workshops

The resulting artwork was displayed at Science Gallery London, London, UK, as part of the Illuminating the Brain Showcase. The piece consists of several coloured boxes seen in Figs. 3 and 4. The graphic designer describes the piece as:

Playing on the tension between art and science, the accuracy and consistency of the box form, is coupled with the play of colour and story. Each box pays tribute to one or more themes explored throughout the project - light, colour, duality, time, control, distance, loss, darkness and memory.

**Fig. 5:**
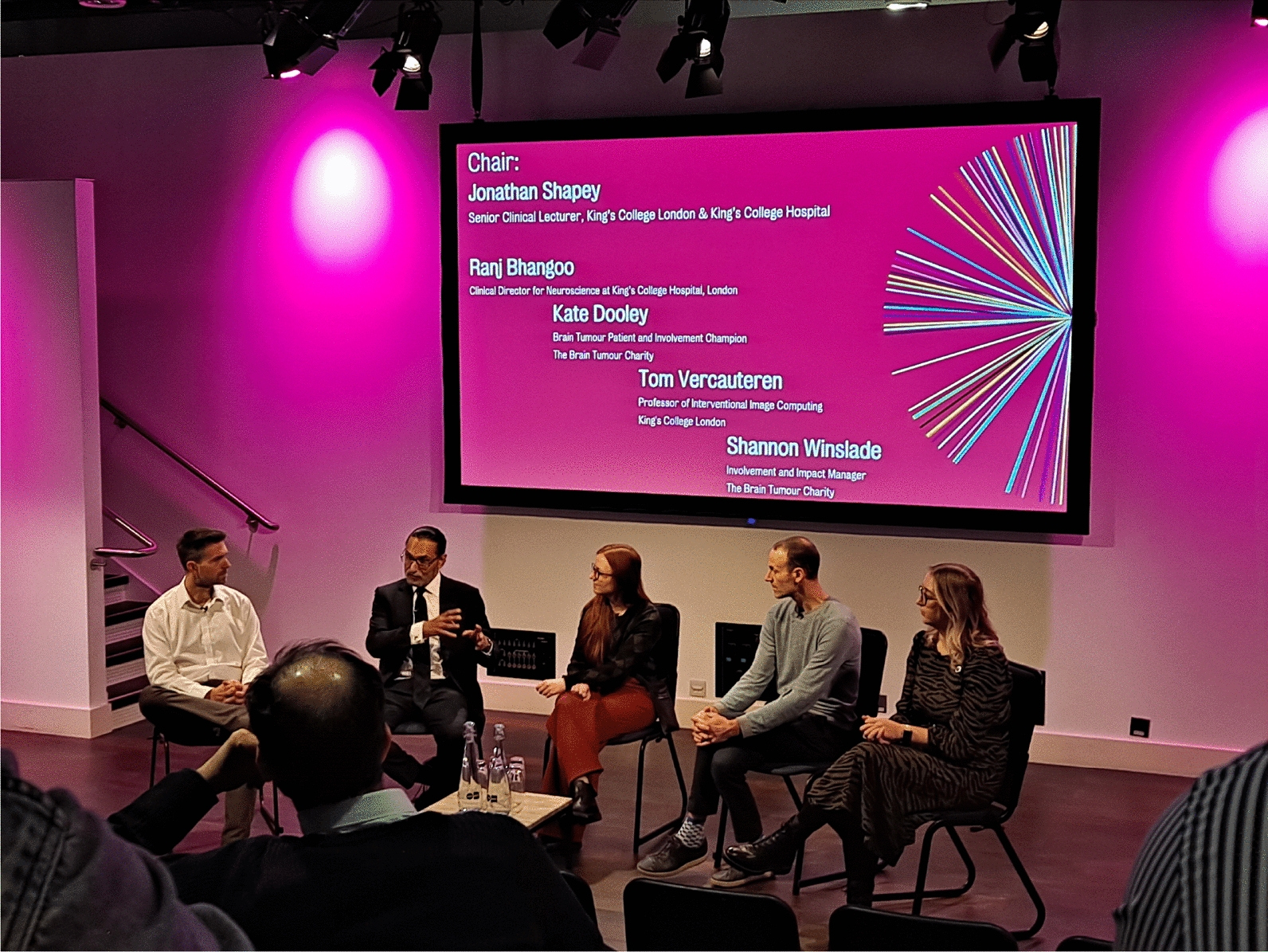
Public questions and answers discussion with our panel of a consultant neurosurgeon, patient representatives from The Brain Tumour Charity the PI from our research studies

As well as displaying the artwork, this showcase event allowed members of the public to attend a talk on the work we are doing and engage with a panel consisting of a consultant neurosurgeon, a patient representative from The Brain Tumour Charity, an involvement and impact champion from the Brain Tumour Charity and the principle investigator (PI) from our research team in a questions and answers session, chaired by one of our project’s senior co-investigators Fig. 5.

Overall, feedback from the public was very positive, exemplified by the following quotes:

I thought the artwork was very thought provoking, especially as someone with lived experience of a brain tumour its the first time I’ve seen an artist attempt to capture how it feels. I was impressed with the overall look of the artwork, and detailed explanations, and being able to walk within it was very powerful. Well done!

I thought the whole event was very well run, looked very professional and it’s a shame if it was only for the one night. I hope many others have been given the opportunity to see the artwork.

## Discussion

This commentary demonstrates our experience of PPI in neurosurgical studies, our methods for incorporating PPI and, importantly, evidence of the clear value added to our work, particularly with reference to research ethics, study participant recruitment and dissemination of our work. As well as this, the group ensured that our work remained patient centred throughout; whilst this is more difficult to specifically quantify, it is no less valuable than the examples outlined above.

Whilst our PPI and engagement work has been a success, there are clear areas for improvement. One of these areas is diversity within our group. Our group has been successful in improving on the literature reported pattern for PPI group participants, as white, middle class and retired [29, 36], with group members across age ranges, social backgrounds and with only one retiree, however we have not been successful with regards to achieving a more ethnically diverse representation within the group. Ultimately, without the limitations imposed by the COVID-19 pandemic at the time of recruitment, we would seek to recruit from different environments that may better reach participants from underrepresented backgrounds, such as places of worship and community centres [37, 38].

As well as this, there were also areas highlighted to improve on with regards to the Illuminating the Brain project. One example was that descriptions from the artwork were not easily accessible to all attendees. To improve upon this, we would ensure that these information sheets are handed to attendees as they enter the showroom. This ensures simultaneous appreciation of the artwork and understanding of its relationship to our research, resulting in a more impactful engagement activity

Taking into account the above, and maintaining our intended objective of providing a simple blueprint for neurosurgical researchers wishing to undertake PPI/E work, we have summarised our recommendations, based on our experiences in Table 2

**Table 2:** Summary of recommendations

Aspect	Recommendation(s)		Limitations
Funding	Budget Precisely	Ensure that involvement and engagement activities are budgeted for separately	None
	Utilise available resources	e.g. NIHR payment guidance for researchers and professionals	Risks becoming prescriptive
Patient Group Participant Recruitment	Recruit in person	e.g. Outpatient clinic	None
	Approach Relevant Charities	Many have PPI experience	May introduce bias towards those already interested in helping with research
	Employ Social Media		Can introduce participant recruitment bias
	Utilise Community Media	e.g. Periodicals for places of worship	Can introduce participant recruitment bias
Group dynamics	Recruit Independent Facilitator	Ensures all participants feel able to contribute and are given the opportunity to do so. Act as a single point of contact for all patient participants. Important for consistency and maintaining relationships. Ensures group stays within scope of patient and researcher expertise.	Requires additional funding and/or specific training
	Recruit Patient Chairperson	Ensures activities are appropriate and not overwhelming. Supports patient participants in their contributions.	May require specific training
Group Activity Planning	Maintain Reciprocal Relationships	Ensure all stakeholders have a voice. Patient chairperson and independent facilitator play a crucial role here.	None
	Promote Co-Learning	Equal learning for both patient participants and researchers	None
	Encourage Partnerships	Ensure participants are financially compensated. Ensure accessibility and diversity are considered.	Funding implications
	Maintain Transparency, Honesty and Trust	Open channel of communication not limited to group meetings. An independent facilitator is helpful here.	None
Engagement Activity	Ensure Research Centred	Ensure the activity and associated materials are accessible and clearly linked to the research question	None

## Conclusion

In conclusion, this commentary underscores the pivotal role of patient and public involvement (PPI) in enhancing the quality and relevance of neurosurgical research. Our experiences highlight the tangible benefits of incorporating PPI and, moving forward, we remain committed to fostering meaningful engagement with patients and the public in our research endeavors, recognising the invaluable contributions they make to advancing neurosurgical knowledge and practice.

## Data Availability

Data sharing is not applicable to this article as no datasets were generated or analysed during the current study.

## References

[1] Jahangiri A, Flanigan PM, Arnush M, Chandra A, Rick JW, Choi S, Chou A, Berger MS, Aghi MK. From bench to bedside: trends in National Institutes of Health funding for neurosurgeons from 1991 to 2015. J Neurosurg. 2019;133(3):865–74. 10.3171/2019.1.JNS181531. 31470404 10.3171/2019.1.JNS181531

[2] ReFaey K, Freeman WD, Tripathi S, Guerrero-Cazares H, Eatz TA, Meschia JF, Carter RE, Petrucelli L, Meyer FB, Quinones-Hinojosa A. NIH funding trends for neurosurgeon-scientists from 1993–2017: biomedical workforce implications for neurooncology. J Neuro-Oncol. 2021;154(1):51–62. 10.1007/S11060-021-03797-5. 10.1007/s11060-021-03797-5 PMC8684039 34232472

[3] Visconti-Lopez FJ, Saal-Zapata G. Global research trends of neurosurgery: a comprehensive bibliometric and visualized analysis of systematic reviews. World Neurosurg. 2023;176:e345–56. 37244520 10.1016/j.wneu.2023.05.061

[4] Deighton AJ, Chhatwal K, Das D. Digital technology: digital tools in neurosurgical pathways: considerations for the future. Future Healthc J. 2022;9(1):67–74. 35372781 10.7861/fhj.2021-0163 PMC8966794

[5] Brett J, Staniszewska S, Mockford C, Herron-Marx S, Hughes J, Tysall C, Suleman R. A systematic review of the impact of patient and public involvement on service users, researchers and communities. Patient. 2014;7(4):387–95. 10.1007/S40271-014-0065-0. 25034612 10.1007/s40271-014-0065-0

[6] Arumugam A, Phillips LR, Moore A, Kumaran SD, Sampath KK, Migliorini F, Maffulli N, Ranganadhababu BN, Hegazy F, Botto-van Bemden A. Patient and public involvement in research: a review of practical resources for young investigators. BMC Rheumatol. 2023. 10.1186/S41927-023-00327-W. 36895053 10.1186/s41927-023-00327-w PMC9996937

[7] Blackburn S, McLachlan S, Jowett S, Kinghorn P, Gill P, Higginbottom A, Rhodes C, Stevenson F, Jinks C. The extent, quality and impact of patient and public involvement in primary care research: a mixed methods study. Res Involv Engagem. 2018;4(1):1–18. 10.1186/S40900-018-0100-8/TABLES/6. 29850029 10.1186/s40900-018-0100-8 PMC5966874

[8] Marston C, Renedo A. Understanding and measuring the effects of patient and public involvement: an ethnographic study. Lancet. 2013;382:69. 10.1016/s0140-6736(13)62494-0.

[9] Wicks P, Richards T, Denegri S, Godlee F. Patients’ roles and rights in research. BMJ. 2018. 10.1136/BMJ.K3193. 30045909 10.1136/bmj.k3193

[10] Marcus HJ, Bennett A, Chari A, Day T, Hirst A, Hughes-Hallett A, Kolias A, Kwasnicki RM, Martin J, Rovers M, Squire SE, McCulloch P. IDEAL-D framework for device innovation: a consensus statement on the preclinical stage. Ann Surg. 2022;275(1):73–9. 10.1097/SLA.0000000000004907. 33856386 10.1097/SLA.0000000000004907 PMC8683254

[11] Mojadeddi ZM, Öberg S, Rosenberg J. Low degree of patient involvement in contemporary surgical research: a scoping review. J Postgrad Med. 2023;69(3):153–8. 10.4103/JPGM.JPGM_83. 37357485 10.4103/jpgm.jpgm_83_23 PMC10394534

[12] Owyang D, Bakhsh A, Brewer D, Boughton OR, Cobb JP. Patient and public involvement within orthopaedic research: a systematic review. J Bone Jt Surg Am. 2021;103(13):51. 10.2106/JBJS.20.01573. 10.2106/JBJS.20.01573 34228669

[13] Lang I, King A, Jenkins G, Boddy K, Khan Z, Liabo K. How common is patient and public involvement (PPI)? Cross-sectional analysis of frequency of PPI reporting in health research papers and associations with methods, funding sources and other factors. BMJ Open. 2022;12(5):063356. 10.1136/BMJOPEN-2022-063356. 10.1136/bmjopen-2022-063356 PMC9131100 35613748

[14] Bagley HJ, Short H, Harman NL, Hickey HR, Gamble CL, Woolfall K, Young B, Williamson PR. A patient and public involvement (PPI) toolkit for meaningful and flexible involvement in clinical trials–a work in progress. Res Involv Engagem. 2016. 10.1186/S40900-016-0029-8. 29062516 10.1186/s40900-016-0029-8 PMC5611579

[15] Absolom K, Holch P, Woroncow B, Wright EP, Velikova G. Beyond lip service and box ticking: how effective patient engagement is integral to the development and delivery of patient-reported outcomes. Qual Life Res. 2015;24(5):1077–85. 10.1007/S11136-014-0909-Z/TABLES/1. 25577498 10.1007/s11136-014-0909-z

[16] Gray-Burrows KA, Willis TA, Foy R, Rathfelder M, Bland P, Chin A, Hodgson S, Ibegbuna G, Prestwich G, Samuel K, Wood L, Yaqoob F, McEachan RRC. Role of patient and public involvement in implementation research: a consensus study. BMJ Qual Saf. 2018;27(10):858–64. 10.1136/BMJQS-2017-006954. 29666310 10.1136/bmjqs-2017-006954 PMC6166593

[17] MacCormac O, Horgan C, Noonan P, Janatka M, Trotouin T, Jacobs J, Wang Z, Bahl A, Elliot M, Segaud S, Waterhouse D, Patel S, Kailaya-Vasan A, Lavrador J, Ashkan K, Ebner M, Ourselin S, Vercauteren T, Shapey J. Real-time intra-operative hyperspectral imaging derived tissue properties in neurosurgery: a first in human case series (IDEAL 1 and 2a). Brain Spine. 2024;4:103594. 10.1016/J.BAS.2024.103594.

[18] MacCormac O, Noonan P, Janatka M, Horgan CC, Bahl A, Qiu J, Elliot M, Trotouin T, Jacobs J, Patel S, Bergholt MS, Ashkan K, Ourselin S, Ebner M, Vercauteren T, Shapey J. Lightfield hyperspectral imaging in neuro-oncology surgery: an IDEAL 0 and 1 study. Front Neurosci. 2023;17:1239764. 10.3389/FNINS.2023.1239764/BIBTEX. 37790587 10.3389/fnins.2023.1239764 PMC10544348

[19] Benedict C, Hahn AL, Diefenbach MA, Ford JS. Recruitment via social media: advantages and potential biases. Digit Health. 2019. 10.1177/2055207619867223. 31431827 10.1177/2055207619867223 PMC6685119

[20] Camberwell Green (Ward, United Kingdom)—Population statistics, charts, map and location. https://citypopulation.de/en/uk/london/wards/southwark/E05011096__camberwell_green/.

[21] Kent population stats in maps and graphs. https://www.plumplot.co.uk/Kent-population.html.

[22] NIHR: PCORI Engagement Rubric. PCORI (Patient-Centered Outcomes Research Institute). Website http://www.pcori.org/sites/default/files/Engagement-Rubric.pdf. Published February 4th 2014, updated October 12th 2016. Accessed 14th March 2024.

[23] Jackson T, Pinnock H, Liew SM, Horne E, Ehrlich E, Fulton O, Worth A, Sheikh A, De Simoni A. Patient and public involvement in research: from tokenistic box ticking to valued team members. BMC Med. 2020;18(1):1–7. 10.1186/S12916-020-01544-7/TABLES/1. 32279658 10.1186/s12916-020-01544-7 PMC7153227

[24] Todd S, Coupland C, Randall R. Patient and public involvement facilitators: Could they be the key to the NHS quality improvement agenda? Health Expect Int J Public Particip Health Care Health Policy. 2020;23(2):461. 10.1111/HEX.13023. 10.1111/hex.13023 PMC7104637 32022356

[25] Biggane AM, Olsen M, Williamson PR. PPI in research: a reflection from early stage researchers. Res Involv Engagem. 2019;5(1):1–9. 10.1186/S40900-019-0170-2/PEER-REVIEW. 31832239 10.1186/s40900-019-0170-2 PMC6865031

[26] Alesandrini KL. Pictures and adult learning. Instruct Sci. 1984;13(1):63–77. 10.1007/BF00051841/METRICS.

[27] NIHR: Payment guidance for researchers and professionals | NIHR (2022). https://www.nihr.ac.uk/documents/payment-guidance-for-researchers-and-professionals/27392.

[28] Staley K. ‘Is it worth doing?’ Measuring the impact of patient and public involvement in research. Res Involv Engagem. 2015;1(1):1–10. 10.1186/S40900-015-0008-5/TABLES/3. 29062495 10.1186/s40900-015-0008-5 PMC5598089

[29] Russell J, Fudge N, Greenhalgh T. The impact of public involvement in health research: What are we measuring? Why are we measuring it? Should we stop measuring it? Res Involv Engagem. 2020;6(1):1–8. 10.1186/S40900-020-00239-W/PEER-REVIEW. 33133636 10.1186/s40900-020-00239-w PMC7592364

[30] Staley K, Barron D. Learning as an outcome of involvement in research: What are the implications for practice, reporting and evaluation? Res Involv Engagem. 2019;5(1):1–9. 10.1186/S40900-019-0147-1/TABLES/2. 30915234 10.1186/s40900-019-0147-1 PMC6416961

[31] Mathie E, Wilson P, Poland F, Mcneilly E, Howe A, Staniszewska S, Cowe M, Munday D, Goodman C. Consumer involvement in health research: a UK scoping and survey. Int J Consum Stud. 2014;38(1):35–44. 10.1111/IJCS.12072.

[32] Friesen P, Lignou S, Sheehan M, Singh I. Measuring the impact of participatory research in psychiatry: how the search for epistemic justifications obscures ethical considerations. Health Expect Int J Public Particip Health Care Health Policy. 2021;24 Suppl 1(Suppl 1):54–61. 10.1111/HEX.12988. 10.1111/hex.12988 PMC8137501 31854081

[33] Festing S, Wilkinson R. The ethics of animal research. Talking Point on the use of animals in scientific research. EMBO Rep. 2007;8(6):526. 10.1038/SJ.EMBOR.7400993. 17545991 10.1038/sj.embor.7400993 PMC2002542

[34] Ibbetson C. Where do Britons stand on animal testing? | YouGov 2021. https://yougov.co.uk/health/articles/39468-where-do-britons-stand-animal-testing.

[35] Tierney S, Dawson S, Boylan AM, Richards G, Park S, Turk A, Babatunde O. Broadening diversity through creative involvement to identify research priorities. Res Involv Engagem. 2021;7(1):1–10. 10.1186/S40900-020-00244-Z/FIGURES/5. 33407929 10.1186/s40900-020-00244-z PMC7787225

[36] Maguire K, Britten N. “How can anybody be representative for those kind of people?’’ Forms of patient representation in health research, and why it is always contestable. Soc Sci Med (1982). 2017;183:62–9. 10.1016/J.SOCSCIMED.2017.04.049. 10.1016/j.socscimed.2017.04.049 28463721

[37] NIHR: Being inclusive in public involvement in health and care research | NIHR, 2021. https://www.nihr.ac.uk/documents/being-inclusive-in-public-involvement-in-health-and-care-research/27365.

[38] Nimmons D, Pigott J, Dowridge W, Ogunleye D, Walters K, Davies N. Ensuring diversity in patient and public involvement in research—BJGP Life. https://bjgplife.com/ensuring-diversity-in-patient-and-public-involvement-in-research/.

